# Acute Necrotizing Encephalitis Due to Influenza B in a Child: A Case Report

**DOI:** 10.7759/cureus.38573

**Published:** 2023-05-05

**Authors:** Prashant Kumar Singh, Rohit Sharma, Chanakya Saini, John Murray, Steven Parrish Winesett

**Affiliations:** 1 Child Neurology, University of Florida Health, Gainesville, USA; 2 Internal Medicine, Geisinger Health System, Wilkes-Barre, USA; 3 Medicine, Even Healthcare, Bengaluru, IND; 4 Neurology, Geisinger Commonwealth School of Medicine, Scranton, USA; 5 Pediatrics, University of Florida Health, Gainesville, USA

**Keywords:** acute necrotizing encephalopathy type 1, cytokine storm syndrome, cauda equina, influenza b, acute necrotizing encephalitis

## Abstract

Acute necrotizing encephalitis (ANE) is a rare and life-threatening form of encephalitis associated with influenza virus and other pathogens. It is characterized by a rapid onset of neurological symptoms and has been linked to a cytokine storm within the brain. We present a unique case of an eight-year-old female with influenza B-associated ANE, involving multiple brain areas including the cerebellum and brainstem and cauda equina involvement. The patient had a rapid neurological deterioration, and MRI findings revealed extensive multifocal areas of abnormal brain parenchyma and inflammation with Guillain-Barre appearance in the cauda equina. To the best of our knowledge, this is the first reported case of ANE with cauda equina involvement leading to neurological deficits. Despite treatment with oseltamivir, steroids, and intravenous immunoglobulins, the patient had poor neurological outcomes, similar to those reported in the literature.

## Introduction

Acute necrotizing encephalitis (ANE) is an extremely rare and catastrophic form of encephalitis that can be caused by influenza virus. The incidence of ANE is not well known, but it is mainly seen in the far east region, particularly in Japan and Taiwan. However, sporadic cases of ANE have been reported worldwide [1]. ANE mostly affects patients ages from 5 months to 11 years with the peak incidence between ages 6 and 18 months [1]. ANE was first described in Japan by Mizuguchi et al. and is believed to be a para-infectious process. Although there is no high-level evidence available, most experts agree that pathology is related to high levels of proinflammatory cytokines with soluble cytokine receptors causing a “cytokine storm” diffusely within the brain [2]. So far, outcome studies have shown significantly poor neurological outcomes with increased morbidity and mortality [3,4]. One study also notes no difference in outcomes in ANE between influenza and non-influenza cases [5]. A scoring system has been proposed by Yamamoto et al. known as the ANE severity score which takes into account shock, brainstem lesion, age> 48 months, platelet count < 100,000, and cerebrospinal fluid (CSF) protein > 60 mg/dl [6]. The total score ranges from 0 to 9 points. A higher score is associated with poor outcomes [6]. We present a case of influenza B-associated ANE in an eight-year-old female affecting multiple areas of the brain including the cerebellum and brainstem. This unique case also demonstrated cauda equina involvement which has not been reported in the literature before.

## Case presentation

An eight-year-old female presented to the emergency department (ED) for evaluation of altered mental status, lethargy, and flu-like symptoms. The fever and vomiting started three days ago prior to admission. The mother decided to take her to a primary care physician where she was diagnosed with Influenza A/B by point-of-care testing. She was sent home with anti-emetics and oseltamivir which was not started by her parents. She subsequently presented to the ED with recurrent emesis, urinary retention, and lethargy. The initial general examination was positive for tachycardia and a dry mucous membrane. Neurological examination showed a GCS of 7 (E1V1M5), pupils were small (2mm) sluggishly reactive to light, and disconjugate gaze (exotropia of OD) was noted. Facial sensation was unable to be adequately assessed. There was intact corneal reflex bilaterally (R<L). No facial asymmetry was noted. Hearing appears to be grossly intact. The patient was able to flex her elbows and shoulders as well as her hips in withdrawal to distal and proximal noxious stimuli (stronger response on the right than the left side), and bilateral upgoing toes. No clonus elicited. Coordination and gait were unable to be formally assessed. The reported family history was negative for any neurological illness.

**Figure 1:**
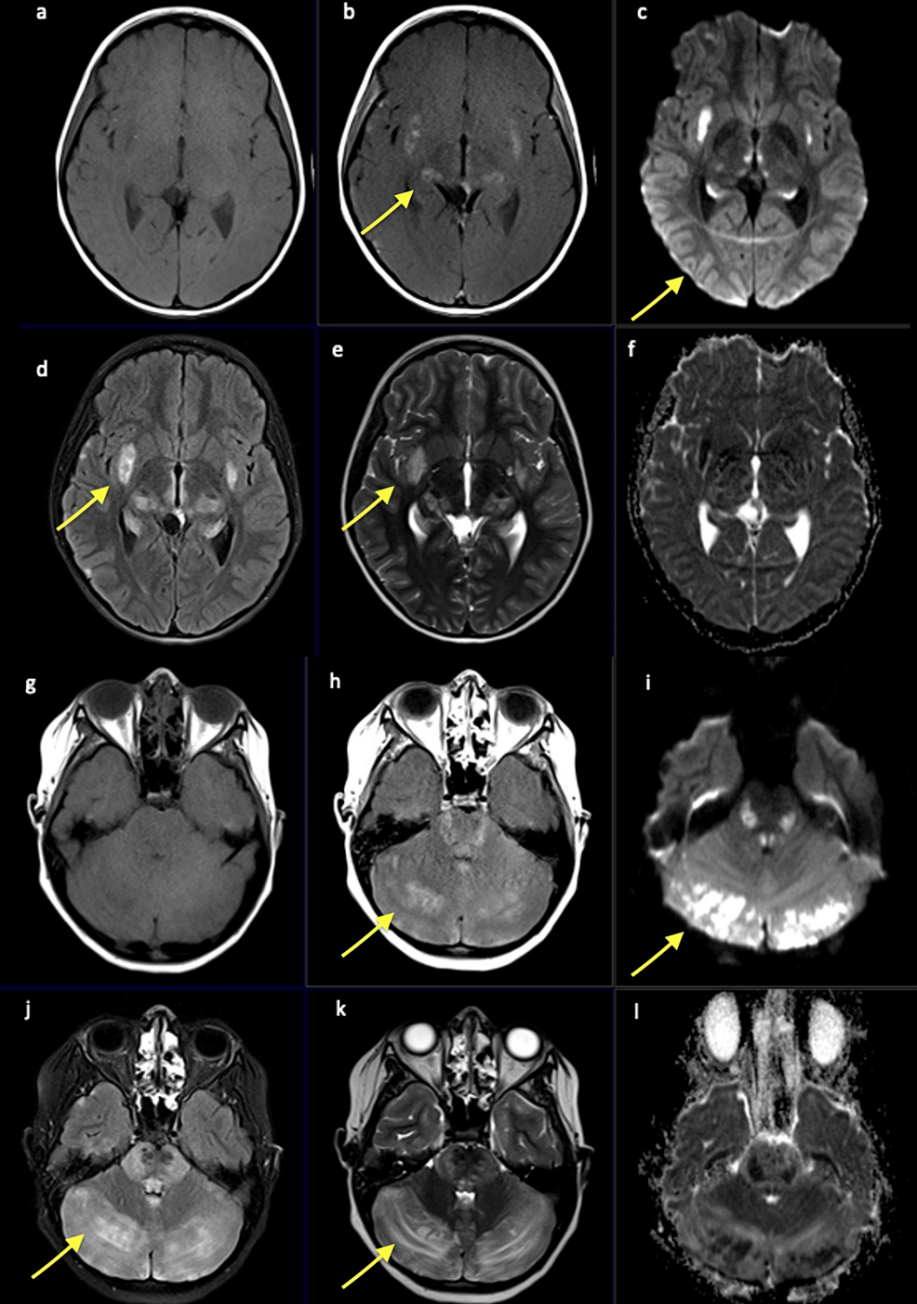
Axial T1-weighted imaging (a-g) and gadolinium-enhanced T1- weighted imaging (b-h), diffusion-weighted imaging (c-i), axial FLAIR-weighted imaging (d-j), T2-weighted imaging (e-k), and apparent diffusion coefficient (f-l) demonstrating multifocal areas of T2/FLAIR hyperintensity (indicated by yellow arrows) with areas diffusion restriction involving the bilateral right greater than left external capsule/lateral putamen, bilateral medial thalami, and amygdala, hippocampus, hypothalamus, midbrain, pons, superior cerebellar peduncle, and cerebellum, vermis, and brainstem. FLAIR: Fluid-attenuated inversion recovery

**Figure 2:**
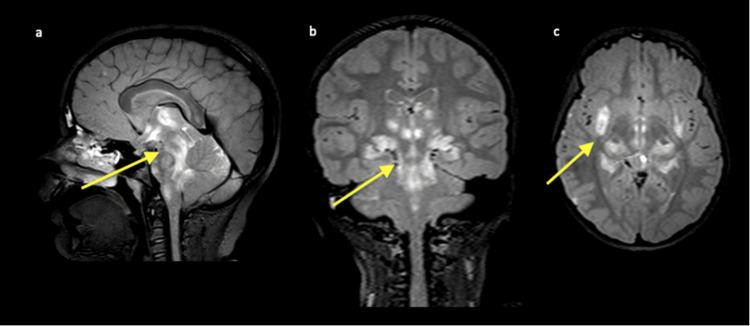
Sagittal (a), coronal (b), and axial (c) 3D FLAIR weighted images demonstrating diffuse brain swelling particularly involving the brainstem and cerebellum with narrowing of the basal cisterns and cerebral sulci as well as multifocal areas of FLAIR hyperintensity (indicated by yellow arrows) involving the lateral basal ganglia, putamen, medial thalamus and hypothalamus, midbrain, pons, medulla, hippocampus, and bilateral cerebellum. FLAIR: Fluid-attenuated inversion recovery; 3D: three-dimensional

Differential diagnoses included infectious meningitis/encephalitis, parainfectious/post-infectious meningoencephalitis, acute disseminated encephalomyelitis, neuromyelitis-optica spectrum disorder (NMOSD), mitochondrial diseases like Leigh syndrome, and lymphoma. The initial workup included a comprehensive metabolic panel, complete blood count, procalcitonin, C reactive protein, and urinalysis, and all were unremarkable. A nasopharyngeal respiratory viral panel (RVP by polymerase chain reaction) was done and was positive for influenza B. Head computed tomography (CT) showed edema throughout both cerebellar hemispheres with mild effacements of the fourth ventricle. No evidence of obstructive hydrocephalus was seen, and there was also noted to have hypodensity lateral to the right putamen. To further evaluate abnormalities from the CT scan, a brain magnetic resonance imaging (MRI) was done with findings of extensive multifocal areas of abnormal brain parenchymal T2/FLAIR hyperintensity with diffusion restriction and patchy enhancement involving the supratentorial brain, pons, basal ganglia, thalamus, brainstem, and cerebellum (Figures 1, 2).

**Figure 3:**
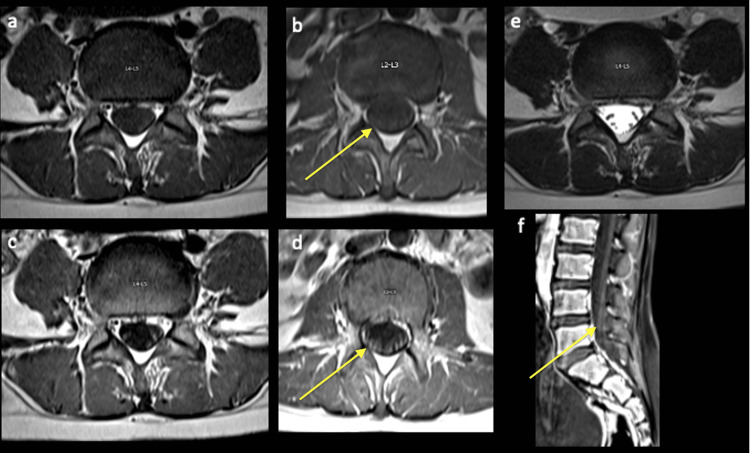
Axial T1-weighted (a- L4-L5, b L2-L3), gadolinium-enhanced T1-weighted (c- L4-L5, d L2-L3), axial T2-weighted (e, L4-L5), and sagittal gadolinium-enhanced T1-weighted (f) images demonstrating mild diffuse thickening and enhancement of cauda equina (enhancement marked by yellow arrows).

MRI findings were compatible with infectious meningitis/encephalitis, parainfectious/post-infectious necrotizing encephalitis, meningitis, and demyelinating diseases like acute disseminated meningoencephalitis. NMOSD was considered. MRI also showed inflammation with Guillain-Barre appearance in the cauda equina likely related to an autoimmune response (Figure 3).

She was admitted to the pediatric intensive care unit (PICU) where she was electively intubated for airway protection given continuous emesis and a degree of lethargy. Further extensive workup included the serum viral panel, ammonia, blood culture, urine toxicology, and lactic acid which were unremarkable. Lumbar puncture showed an opening pressure of 27 cm of H_2_O and a closing pressure of 19 cm of H_2_O. The CSF profile showed a white blood cell count of 1 cell/microL, a red blood cell count of 0 cells/microL, protein of 181 mg/dL, and glucose of 76 mg/dL with serum glucose of 109, and a negative meningoencephalitis panel (see Table 1 for full laboratory values). Her initial treatment consisted of ceftriaxone, vancomycin, and acyclovir due to the concern for infectious meningoencephalitis. She was treated with high-dose oseltamivir and steroids for 10 days and two days of intravenous immunoglobulins (IVIG). At the time of discharge to a rehabilitation facility, the patient was nonverbal, unable to walk, gastric tube-dependent, and requiring a bladder scan and straight catheterization for urinary retention. Anti-N-methyl-D aspartate receptor and voltage-gated potassium channel antibodies were negative. A month after discharge, the patient tested positive for the acute necrotizing encephalopathy type 1 (ANE1) gene (pathogenic RAN Binding Protein 2 also known as RANBP2 mutation by Next Generation Sequencing via Fulgent Laboratories). 

**Table 1:** Laboratory values for the case. WBC: White blood cell, RBC: red blood cell, PCR: polymerase chain reaction, CSF: cerebrospinal fluid

Laboratory test	Hospital day	Reference range for age (unit)	Laboratory result
WBC count	1	4.5 – 13.5 (x 10^9^ cells/ L)	10.2
Hemoglobin	1	11.5 – 13.5 (g/L)	11.6
RBC	1	3.90 – 5.30 (x 10^12^ cells/L)	4.28
Neutrophil %	1	40.0 – 80.0 %	88.7 %
C-reactive protein	1	0.00 – 5.00 (mg/L)	3.04
Procalcitonin	1	0.00 – 0.60 9ng/mL)	0.20
Respiratory PCR panel (nasopharyngeal)	1	Negative	Influenza B positive detected by PCR
Blood culture	1	Negative/no growth	Negative/no growth
Ammonia	2	18 – 72 µ/dL	59
Lactic acid	2	0.3 – 1.5.mg/dL	< 0.5
Herpes simplex virus 1/2 DNA PCR	2	Negative	Negative
CSF cell count	2	WBC count 0 - <5/mm RBC count 0- <5/mm	WBC count – 1 RBC - 0
CSF glucose	2	40 – 70 mg/dL	74
CSF protein	2	15 – 45 mg/dL	181
CSF gram stain	2	Negative	Negative
CSF enterovirus	2	Negative	Negative
CSF meningitis/encephalitis panel	3	Negative	Negative
West Nile virus panel	4	Negative	Negative
Arbovirus panel	5	Negative	Negative
CSF mycoplasma (PCR)	5	Negative	Negative
CSF respiratory semi-quantitative PCR	15	Negative	Negative
Acute necrotizing encephalopathy type 1 gene	49	Negative	An autosomal dominant mutation in RANBP2 N_M006267.4 Variant c.1754C>Tp.Thr585Met, Zygosity Heterozygous Classification pathogenic

## Discussion

ANE is a serious, life-threatening pathology that is potentially underreported and under search. In 1995, it was first reported by Mizuguchi et al [1]. While data are limited, one study suggests that ANE is present in up to 4% (of 983) of children admitted with acute encephalopathy [7]. It most commonly occurs due to viral infections such as influenza A/B, Herpes Simplex Virus (HSV), and human Herpes Virus 6 (HHV-6) [8]. Bacterial causes are less common, but case reports from mycoplasma pneumonia-causing ANE have been reported. In the 1990s, it was initially thought to be predominantly present in East Asia due to multiple case reports coming out from Japan leading to speculation that East-Asian ethnicity could be a risk factor. Later on, ANE has been reported globally including in the UK, Australia, Turkey, Chile, Hong Kong, etc. [3, 9-12]. It is important to recognize the devastating neurological complication of a common infection such as influenza which could further add to the importance of the flu vaccine.

We report an unusual case of an eight-year-old female with influenza B-positive ANE with typical findings of extensive multifocal areas of abnormal brain parenchyma involvement including the supratentorial brain, ganglia, thalamus, brainstem, and cerebellum. The patient had MRI findings of mild diffuse enhancement of cauda equina with a Guillain-Barre-like appearance which might have led to urinary retention requiring intermittent catheterization. To our knowledge, we present the first case of ANE along with cauda equina involvement leading to neurological deficits. Rapid neurological deterioration with typical radiological findings was noted to be similar to previously reported cases.

Diagnoses are based on clinical findings of altered mental status, seizure, and rapidly developing coma with pathognomonic radiological findings of necrosis of bilateral thalami and other brain regions including brainstem tegmentum, cerebral white matter, internal capsule, putamen, and cerebellum [1]. Laboratory findings such as elevated CSF protein are common. Other reported cases have generally noted CSF pleocytosis which was absent in our case. Poor prognostic factors include elevated aspartate transaminase (AST > 500), hypoglycemia, hematuria/proteinuria, and the use of diclofenac sodium [13].

Pathogenesis is unclear but is largely directed toward cytokine storm which was hypothesized due to the presence of high cytokines levels particularly interleukin-6 (IL-6) and tumor necrosis factor- α both in serum and CSF [9,14]. Researchers have previously identified a variant of the RANBP2 gene which is associated with familial acute necrotizing encephalopathy [15]. This mutation was positive in our case. This leads us to support the hypothesis that patients with ANE could have increased susceptibility to immune dysfunction leading to cytokine storm, where susceptibility is likely coming from genetics [10]. 

ANE is historically associated with poor prognosis high mortality and significant neurological morbidity up to 54-56.2 % [10,16]. No clear data are available for long-term sequelae of survivors. An Australian study noted that poor prognosis including death was co-related with MRI findings such as diffusion restriction vouching for the importance of neuroimaging particularly with MRI [10].

Currently, no high-quality data including clinical trials are available owing to the rarity of the disease. Treatments reported in the literature include corticosteroids, IVIG, and plasmapheresis. The Infectious Diseases Society of America suggests using oseltamivir [14]. One case from the UK reported the use of intravenous zanamivir for H1N1-associated ANE with complete recovery by day 8 [17]. Another case series have suggested the use of early use of IL-6 inhibitor such as tocilizumab with favorable outcomes [18]. Hypothermia along with anti-cytokine agents has also been previously proposed [19]. Studies have shown that mortality rates from ANE can range from 30 to 40%. For instance, patients with the RANBP2 mutation have been reported to have a mortality rate of 30% [20], while a single institution in Korea reported a mortality rate of 40% among ANE patients [21].

## Conclusions

ANE is an under-studied diagnosis with unclear pathogenesis. The case presented here raises concern that influenza-associated ANE can also involve cauda equina leading to neurological symptoms. Outcomes, as in the majority of previously reported cases, were poor in terms of neurological morbidity. While it is difficult to perform a randomized trial and with no proven treatment, early use of IL-6 inhibitors along with corticosteroids, IVIG, and plasmapheresis could be advocated based on previous data.
